# Blood Lost: A Retrospective Review of Blood Wastage from a Massive Transfusion Protocol in a Tertiary Paediatric Hospital

**DOI:** 10.3390/children9121799

**Published:** 2022-11-23

**Authors:** Debbra Chong, Joyce Ching Mei Lam, Xun Yi Jasmine Feng, Mui Ling Heng, Yee Hui Mok, Li-Wei Chiang, Kee Chong Ng, Yong-Kwang Gene Ong

**Affiliations:** 1 Haematology Oncology Service, Department of Paediatric Subspecialties, KK Women’s and Children’s Hospital, Singapore 229899, Singapore; 2 Department of Pathology and Laboratory Medicine, KK Women’s and Children’s Hospital, Singapore 229899, Singapore; 3 Department of Emergency Medicine, KK Women’s and Children’s Hospital, Singapore 229899, Singapore; 4 Children’s Intensive Care Unit, Department of Paediatric Subspecialties, KK Women’s and Children’s Hospital, Singapore 229899, Singapore; 5 Department of Paediatric Surgery, KK Women’s and Children’s Hospital, Singapore 229899, Singapore

**Keywords:** massive transfusion, wastage

## Abstract

Background: The paediatric massive transfusion protocol (MTP) is activated in the paediatric population for both trauma and non-trauma related indications. While it helps to improve the efficiency and efficacy of the delivery of blood products, it can also result in increased wastage. We aimed to evaluate the wastage rates from our paediatric MTP activations from 2013 to 2018. Method: As part of an audit, we retrospectively reviewed the records of the paediatric patients who had MTP activations. We collected the following data: reason for MTP activation, weight of patient, number of cycles of MTP required, blood products used, blood products wasted, deviation from our institution’s recommended MTP blood product ratio, and reason for wastage. Result: We had 26 paediatric MTP activations within the audit period. There was an overall wastage rate of 1.5%, with wastage occurring in 3 out of 26 patients. The reason for all wastage was demise of the patient. Most patients’ transfusion ratios deviated from our institution’s MTP protocol. Conclusion: Our wastage rates are low likely because of clear MTP activation guidelines and a flexible MTP workflow.

## 1. Introduction

The massive transfusion protocol (MTP) aims to deliver a pre-defined ratio of blood products, consisting of red cell concentrate (RCC), fresh frozen plasma (FFP), and platelets to a patient. This is to achieve haemostasis in a massively haemorrhaging patient with minimal use of crystalloids and without causing haemodilution. The use of an MTP has been shown to reduce mortality and improve outcomes in the adult population, which may be due to optimising the transfusion ratio of fresh frozen plasma and platelets to red blood cells [1]. In the paediatric population, there is no consensus on the criteria for initiating an MTP or the ideal ratio of blood products to be transfused. However, a commonly used criterion for the initiation of a paediatric MTP is transfusion of more than 40 mL/kg of blood products in the first 24 h [2,3,4].

The KK Women’s and Children’s Hospital (KKH) is a tertiary hospital with 445 paediatric beds, with an average of 400 paediatric emergency visits per day. The paediatric MTP was established in KKH in July 2013.

With the implementation of an MTP in our institution, we were interested in our blood product wastage. There is evidence of increased wastage of blood products, especially fresh frozen plasma (FFP), after the introduction of an MTP [5,6]. Wastage is anticipated in any MTP, as the desired benefit of an MTP is to be able to deliver a set ratio of blood products to the activation site as quickly as possible. Overactivation and, later, failure to terminate the MTP results in blood products being prepared unnecessarily and hence wasted. With the use of blood packs, wastage arises when not all the components are used. There have not been many studies describing blood product wastage in an MTP, and there is even less information on wastage in the paediatric population. However, this is an important topic as blood products are a precious resource, and paediatric transfusion has an increased potential for wastage due to the wide age and weight ranges. This paper therefore aims to look at the wastage of blood products in paediatric MTP activations in KKH from 2013 to 2018.

## 2. Materials and Methods

The data presented in this paper were obtained as part of a retrospective audit of MTP activations from 2013 to 2018, and thus approval from the Institutional Review Board (IRB) was not required, as per the institutional guidelines for both data collection and publication.

The criteria for paediatric MTP activation in our institution are as follows: a patient with ongoing haemorrhagic shock who has received 40 mL/kg of non-blood product fluid boluses or 20 mL/kg of blood products. MTP is activated by the onsite physician, who has to call the blood bank’s dedicated phone line to inform the blood bank of the activation.

There are four subsets in our paediatric MTP based on the weight of the patient, namely: Alpha (patients less than 10 kg), Bravo (patients 10 kg to less than 25 kg), Charlie (patients 25 kg to less than 40 kg), and Delta (patient 40 kg and above). Physicians will need to inform the blood bank which weight subset the patient belongs to when they call to activate MTP.

If not already done, a pre-transfusion blood sample from the patient will be sent for a group and cross match, and baseline laboratory investigations consisting of full blood count, ionised calcium, coagulation profile, and arterial blood gas.

In our institution, we used blood packs, which are a pre-designed set of blood products in a specific ratio that will be prepared and sent to the clinical site during an MTP activation. In Singapore, blood packs are used to facilitate communication with the blood bank and for the blood bank to anticipate and quickly prepare the required blood products [7]. The individual components of blood packs differ according to the institution.

**Figure 1:**
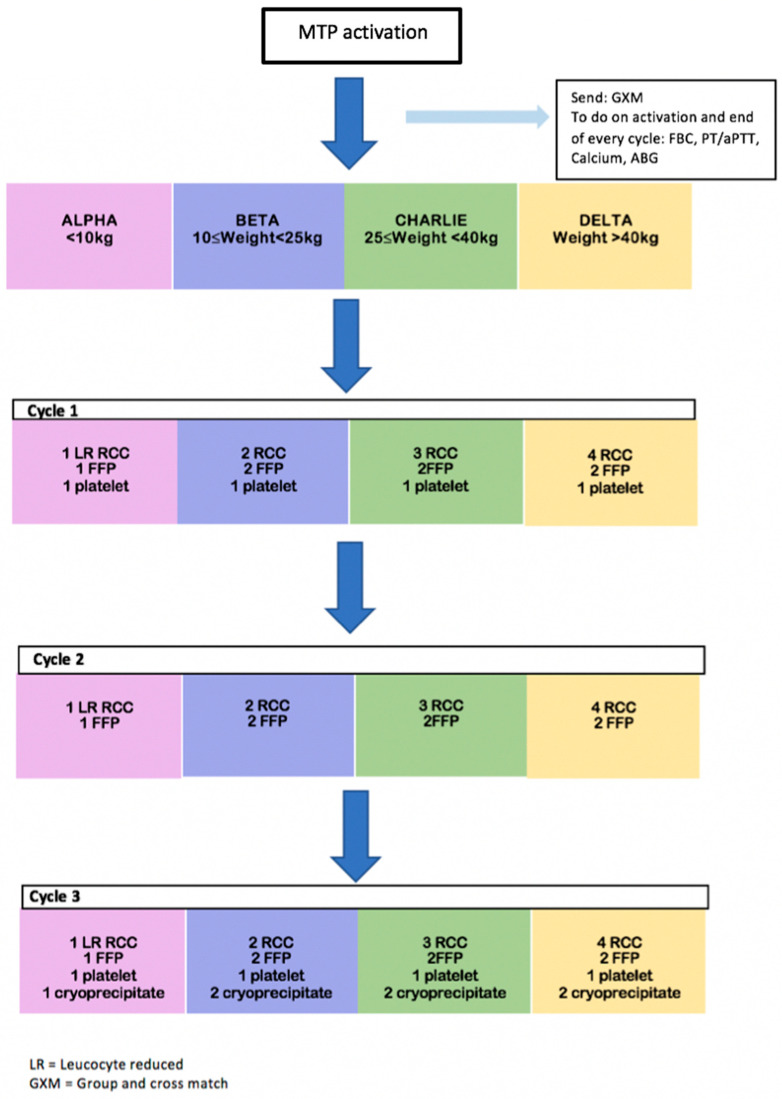
Composition of blood products for each paediatric MTP cycle.

Blood packs will be prepared according to the patient’s weight group. The composition of the various blood packs is shown in Figure 1.

If there is an available group and cross match prior to the MTP activation, blood-group-specific blood products will be issued. Otherwise, emergency blood will be issued. We issue group O Rh(D) positive RCC for Chinese and Malay patients and O Rh(D) negative RCC for Indian patients and patients of other races. All patients receive group AB Rh(D) positive FFP and AB Rh(D) positive apheresed platelets.

After the blood pack in cycle 1 has been prepared and delivered to the MTP activation site, the blood bank will contact the site to confirm whether cycle 2 is needed and whether to go ahead with the preparation of blood products in cycle 2. The same happens for cycle 3.

The number of units of blood products provided for each patient group and each cycle of the MTP is shown in Figure 1 below.

At the end of every cycle, the full blood count, coagulation profile, blood gas, and calcium level are again repeated. If the bleeding has been controlled, the MTP is deactivated by the attending physicians.

Wastage is defined as any blood product that a physician has requested, but is not transfused to the patient, and cannot be reassigned to another patient. If blood products are unused but are returned to the blood bank within 30 min of initial dispatch and are within an optimum temperature (RCC and FFP under 10 degrees and platelets between 20 to 24 degrees), they can be reassigned to another patient and are not considered wasted.

## 3. Results

From 2013 to 2018, the massive transfusion protocol for children was activated for 26 patients. The most common site of MTP activation was from the Children’s Intensive Care Unit (CICU), with 10 activations in this time period (38.5%). There were nine activations (34.6%) from the Children’s Emergency (CE) and seven (26.9%) from the operating theatres (OT). Fifteen activations (57.7%) were for surgery-related bleeding, seven (26.9%) for trauma-related causes, and four (15.4%) were for bleeding related to other medical conditions. The medical conditions associated with bleeding in our audit were disseminated intravascular coagulation (DIC) and, in one patient, bleeding post-thrombolytic therapy.

As per Table 1 below, there were seven patients who had only one cycle activated prior to the cessation of MTP, nine with two cycles, and ten with three cycles. Wastage of blood products occurred in three patients (11.5% of patients) (Table 2).

In the 6 years since the introduction of the MTP, a total of 323 units of blood products were dispatched from the blood bank during this period. These included 168 units of RCC, 43 units of platelets, 77 units of FFP, and 35 units of cryoprecipitate.

In total, two units of RCC and three units of FFP were wasted, which put the overall wastage at 1.5%. Wastage occurred in 3 out of 26 patients for whom the MTP was activated.

The reason for wastage was recorded, and for all three patients it was due to the demise of the patient. When unused blood products were returned to the blood bank they were either out of the temperature range or were returned more than 30 min after issue. Therefore, all unused blood products were wasted.

In addition to the wastage of blood products, we also looked at whether patients actually received blood products in the predefined ratios, as per our MTP.

As seen in Table 3, most of our patients did not receive the blood products according to the predefined ratios of our institution’s MTP. The deviation from the protocol was at the managing physician’s discretion. Only two out of seven patients in the Alpha subgroup (28.5%), one out of seven patients in the Charlie subgroup (14.2%), and one out four patients (25%) in the Delta subgroup received blood products according to the predefined ratios.

As seen in Table 4, in cycle 1 of our MTP protocol, three out of seven patients received blood products in the recommended ratio, but only one out of nine patients (11.1%) in cycle 2 and none in cycle 3.

When blood products were not used in the predefined ratio, we found that FFP was frequently omitted in favour of additional PCT.

In terms of mortality rates in our audit, 5 out of 26 patients (23.8%) died at 24 h post MTP activation, and, in total, 13 out of 26 patients (50%) died prior to discharge.

## 4. Discussion

The evidence for paediatric MTPs is limited compared with the adult population and the ideal blood product ratio in a paediatric MTP is not well established [4,8,9]. However, the availability of an MTP can be beneficial as it provides a workflow that enhances consistent communication and coordination between the clinical staff and the blood bank. It can also guide the laboratory and clinical monitoring of a massively bleeding patient, which can help to improve patient care. For the above reasons, we still see a role for an MTP in any paediatric institution, despite the limited evidence. However, wastage may potentially follow the implementation of an MTP, and many centres have reported an increase in blood wastage rates following its implementation [10,11].

The urgency of the medical condition of patients who require MTP demands rapid preparation and delivery of blood products. This process requires careful coordination and communication, the failure of which can result in blood products being wasted. Some degree of wastage is tolerated and is balanced with the need for the rapid preparation of blood products. A zero-waste MTP is unlikely to be efficient enough in a massively haemorrhaging patient. However, this must be balanced against excessive blood product wastage, especially as blood products are limited resources. According to adult studies, up to 61% of blood products issued to a massively bleeding patient may not be transfused [7]. Common causes of wastage cited in the literature include over-activation and late deactivation of the MTP [12]. Furthermore, upon returning the product to the blood bank, the products are often outside of acceptable temperature ranges, making it unsafe for it to be diverted to other patients [6]. Wastage rates in adult studies are reported to range from 2–7% [11,13]. Our institution’s wastage rate is at 1.5%, which is a comparatively low figure.

There was no wastage in cycle 1 of our MTP, and wastage only occurred in cycles 2 and 3. In cycles 2 and 3, the reason for wastage was due to the death of the patients, thus the blood products were not transfused. For the unused blood products, by the time they were returned to the blood bank, they were either outside of the optimal temperature range or they were returned after 30 min and thus had to be disposed of.

Our relatively low wastage rates may be attributable to a few reasons. Firstly, we noticed that there was no wastage in cycle 1 of the MTP. This suggests that the overactivation of the MTP is not a major concern in our institution, and this may be because there are clear guidelines for activation of the MTP. Wastage is reduced when there are clear guidelines and indications for activation of the MTP. However, we acknowledge that this guideline should not be overly prescriptive, to ensure that we do not exclude patients who will benefit from the MTP.

A second reason for the low wastage rates is the close communication between the blood bank and the MTP activation site. The blood bank calls the MTP site prior to the preparation of the next cycle of blood products to confirm its necessity. In addition, close communication between the two parties allows for flexibility during the resuscitation process.

Physicians are able to request blood products that they require and also inform the blood bank to cease the preparation of blood products that are not required as the clinical situation changes. This communication allows for the targeted preparation and delivery of blood products, thus reducing wastage. Communication that is initiated by the blood bank is especially crucial for reducing wastage as resuscitation sites are frequently busy and administrative processes such as terminating the MTP may be overlooked.

Finally, our weight-based activation of the MTP also plays a role in reducing the rate of wastage. As the MTP packs are prepared and delivered to the site of activation based on the patient’s weight, fewer or smaller units are prepared for smaller paediatric patients. Hence, when there are products not transfused due to circumstances such as the demise of the patient, there are fewer units lost for a patient in a lower weight category.

Even with a low wastage rate, our mortality rates are similar to the existing literature, which is quoted to range from 33.3–59% at 24 h and 17–59% at 30 days [14,15,16].

We acknowledge that our adherence to the blood product ratio of our paediatric MTP is low. This is especially prominent in cycles 2 and 3 of our MTP. We postulate that this may be because of changes in the clinical status of patients as they progressed through the later cycles of the MTP, whereby their blood product needs may have deviated from what is predefined in the standard protocol.

There are limitations to our audit, with it principally being a retrospective, single centre experience with small numbers of MTP activations. Some of our measures to limit wastage may be difficult to implement in a higher volume centre with larger numbers of MTP activations. However, our audit highlights some considerations for helping to reduce the wastage rates of our valuable blood products.

## 5. Conclusions

Based on our experience, we advocate for a weight-based MTP activation protocol with blood packs for reducing waste and for efficiency. There are other interventions that can be taken to reduce the wastage rate, including ensuring clear communication for appropriate activation, request for products, and timely deactivation. Appropriate storage of blood products is also a very important factor. It is not realistic to achieve zero wastage, but it is certainly feasible to limit unnecessary wastage.

This is the summary of our institution’s experience with paediatric MTP activations over 6 years. The number of MTP activations we have is small, but carries promising results. We believe that our methods merit further consideration and study.

## Figures and Tables

**Table 1:** Wastage according to the number of cycles activated.

Total Cycles Activate	Number of Patients (%)	Number of Patients Who Had Wastage
1	7 (26.9%)	0
2	9 (34.6%)	2
3	10 (38.5%)	1

**Table 2:** Blood products (in terms of units) wasted by patient category.

Product Type	Alpha (<10 kg)	Bravo (10 to < 25 kg)	Charlie (25 to < 40 kg)	Delta (≥40 kg)	Wastage (% of Total Dispatched)
RCC	1	1	0	0	1.20%
Platelet	0	0	0	0	0
Fresh frozen Plasma	0	1	0	2	3.90%
Cryoprecipitate	0	0	0	0	0
Total	1	2	0	2	1.50%

**Table 3:** Number of patients in each weight subgroup and number who received blood products in the predefined ratios.

	Number of Patients	No. of Waves Activated			Number Who Received Products According to MTP Ratios	Percentage
		1	2	3		
Alpha (<10 kg)	7	3	4	0	2	28.50%
Bravo (10 ≤ 25 kg)	8	1	3	4	0	0%
Charlie (25 ≤ 40 kg)	7	2	1	4	1	14.20%
Delta (≥40 kg)	4	1	1	2	1	25%

**Table 4:** Number of patients who received blood products in the predefined MTP ratios according to the cycle number.

	Number of Patients	No. Who Received Products According to MTP Ratios	Percentage
Cycle 1	7	3	42.90%
Cycle 2	9	1	11.10%
Cycle 3	10	0	0%

## Data Availability

Not applicable.
